# Supplementary material for: Tweet success? Scientific communication correlates with increased citations in Ecology and Conservation
Source: PeerJ. 2018 Apr 12;6:e4564. doi: 10.7717/peerj.4564 (PMC5899883; doi:10.7717/peerj.4564)
Supplement: Supplemental Information 1 [file peerj-06-4564-s001.doc]

**Supplementary Materials**

**Supplementary 1**

**
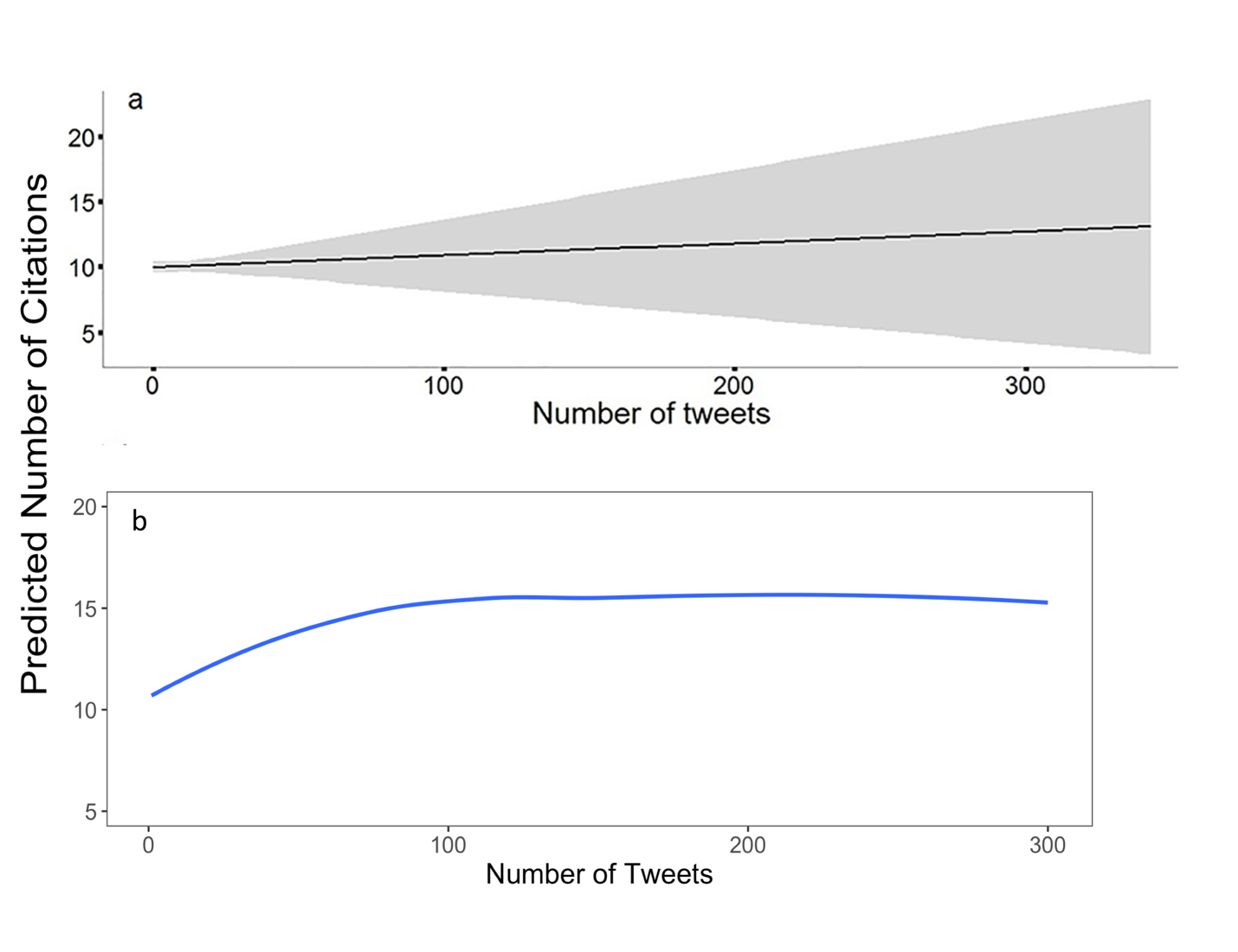
**

**Fig S1.** Predicted number of citations per conservation and ecology article based on the number of tweets from Twitter that article received for A: Peoples et al. (2016) [1], and B: This paper.

**REFERENCES**

1. Peoples BK, Midway SR, Sackett D, Lynch A, Cooney PB. 2016. Twitter predicts citation rates of ecological research. PLoS One 11(11):e0166570 doi:10.1371/journal.pone.0166570
